# An implementation framework and a feasibility evaluation of a clinical decision support system for diabetes management in secondary mental healthcare using CogStack

**DOI:** 10.1186/s12911-022-01842-5

**Published:** 2022-04-14

**Authors:** Dipen Patel, Yamiko J Msosa, Tao Wang, Omar G Mustafa, Siobhan Gee, Julie Williams, Angus Roberts, Richard JB Dobson, Fiona Gaughran

**Affiliations:** 1 Department of Biostatistics and Health Informatics, Institute of Psychiatry, Psychology and Neuroscience, King’s College London, De Crespigny Park, London, SE5 8AB UK; 2 National Institute for Health Research, Maudsley Biomedical Research Centre, South London and Maudsley National Health Service Foundation Trust, De Crespigny Park, London, SE5 8AB UK; 3 Department of Psychosis Studies, Institute of Psychiatry, Psychology and Neuroscience, King’s College London, De Crespigny Park, London, SE5 8AB UK; 4 South London and Maudsley NHS Foundation Trust, London, UK; 5 Department of Diabetes, King’s College Hospital NHS Foundation Trust, Denmark Hill, London, SE5 9RS UK; 6 Centre for Education, Faculty of Life Sciences and Medicine, King’s College London, London, UK; 7 Health Service and Population Research Department, Centre for Implementation Science, King’s College London, London, UK; 8 Institute for Health Informatics, University College London, London, UK; 9 Health Data Research UK London, University College London, London, UK

**Keywords:** Alerting, Clinical decision support, CogStack, Diabetes, EHealth, Pre-diabetes, Monitoring

## Abstract

**Background:**

Improvements to the primary prevention of physical health illnesses like diabetes in the general population have not been mirrored to the same extent in people with serious mental illness (SMI). This work evaluates the technical feasibility of implementing an electronic clinical decision support system (eCDSS) for supporting the management of dysglycaemia and diabetes in patients with serious mental illness in a secondary mental healthcare setting.

**Methods:**

A stepwise approach was taken as an overarching and guiding framework for this work. Participatory methods were employed to design and deploy a monitoring and alerting eCDSS. The eCDSS was evaluated for its technical feasibility. The initial part of the feasibility evaluation was conducted in an outpatient community mental health team. Thereafter, the evaluation of the eCDSS progressed to a more in-depth in silico validation.

**Results:**

A digital health intervention that enables monitoring and alerting of at-risk patients based on an approved diabetes management guideline was developed. The eCDSS generated alerts according to expected standards and in line with clinical guideline recommendations.

**Conclusions:**

It is feasible to design and deploy a functional monitoring and alerting eCDSS in secondary mental healthcare. Further work is required in order to fully evaluate the integration of the eCDSS into routine clinical workflows. By describing and sharing the steps that were and will be taken from concept to clinical testing, useful insights could be provided to teams that are interested in building similar digital health interventions.

## Background

People with serious mental illnesses (SMI) such as schizophrenia, bipolar affective, and schizoaffective disorders have a significantly reduced life expectancy in comparison with the general population [1, 2]. These groups have higher rates of cardiovascular disease (CVD) risk factors such as central obesity, high blood pressure, raised cholesterol levels, and raised blood sugar levels compared with the general population [2]. Improvements to the primary prevention of physical health illnesses like diabetes in the general population have not been replicated to the same extent in people with SMI [3].

Diabetes refers to a group of metabolic disorders characterised by a high blood sugar level over a prolonged period of time, and is most commonly subdivided dependent upon aetiology into Type 1, Type 2, and Gestational diabetes [4, 5]. Type 1 diabetes is caused by an immune-associated or directly immune-mediated destruction of insulin-producing pancreatic $\beta$ cells [6, 7]. Type 2 diabetes results from impaired insulin secretion, insulin resistance or a combination of both [5]. Gestational diabetes is characterised by any degree of glucose intolerance with onset or first recognition during pregnancy [8]. If left untreated or poorly managed, diabetes can lead to various long-term health complications including cardiovascular disease, stroke, chronic kidney disease, foot ulcers, retinopathy and peripheral neuropathy [4, 9]. Diabetes accounts for approximately 10% of healthcare resources in the United Kingdom (UK), and this is set to rise to 17% with an estimated cost of 39.8 billion Great British Pounds (GBP) by 2035 when direct healthcare costs and indirect costs on productivity are taken into account. [10]

Rates of diabetes in the South London area of the UK amongst people with a diagnosis of established psychosis are 20% with a further 30% evidencing dysglycaemia which refers to raised blood sugar levels [11]. Prevalence of diabetes mellitus and abnormal glucose metabolism are known to be higher in the inpatient psychiatric setting compared to the general population [12]. Furthermore, rates of dysglycaemia double in the first year after a first psychotic episode, creating a unique window for prevention strategies to address these risks as early as possible [13]. Diabetes outcomes are poorer in these groups, such that people with schizophrenia with co-occurring diabetes are at increased risk of excess mortality, including post-complication mortality [14].

A key inequality in healthcare provision in patients with SMI is the less than adequate assessment and treatment of physical health conditions such as diabetes. In an attempt to improve the physical health care of people with SMI and close the life expectancy gap a number of evidence based clinical guidelines and policies have been published over the past decade [15–17]. Unfortunately there remains significant variation in the implementation of these guidelines and recommendations for mental health care services, as outlined by the National Audit of Schizophrenia (NAS) [18]. A retrospective audit of people diagnosed with schizophrenia or schizoaffective disorder revealed that among those with high blood sugar, there was recorded evidence of only 53.5% receiving an appropriate intervention and among those with dyslipidaemia, this was only 19.9% [19]. Another study also found that people with SMI and diabetes were not receiving standard care in glucose monitoring or appropriate access to specialist diabetes services when admitted to a psychiatric unit [20].

Globally, studies evaluating the provision of care by clinicians have revealed that there is a sub-optimal uptake of clinical guidelines in actual practice [21, 22]. The underlying factors for this are complex and occur at a combination of patient, clinician and system levels [21, 23]. Barriers to uptake of clinical guidelines include lack of clinician knowledge or confidence in skills, while behavioural theories suggest that performance can be influenced by external stimuli such as reminders and feedback [21, 23–25]. Noting that SMI patients have a high risk of CVD factors and that there is typically a sub-optimal uptake of clinical guidelines, there is a need for more targeted and clinically-informed interventions that improve the standard of physical healthcare screening and interventions offered to people with SMI across both primary and secondary care settings. Assessing the physical health of SMI patients when they are admitted under mental health services offers an opportunity to identify risk factors for developing conditions such as cardiovascular disease or diabetes and provide advice and support on services that can be accessed in hospital and in the community.

Digital health solutions can improve delivery of care through the application of tools such as clinical decision support systems [26]. Electronic clinical decision support systems (eCDSSs) are digital-enabled tools and interventions that are designed to aid directly in clinical decision making. [27, 28]. These eCDSSs can be used to translate clinical data and knowledge from Electronic Health Record (EHR) ecosystems into patient-specific recommendations that can be readily used by clinicians at the point-of-care using appropriate monitoring and alerting tools [27, 29]. A point-of-care monitoring and alerting eCDSS can alert a clinician to relevant clinical information such as a drug-to-drug interaction, an order reminder, or a critical laboratory test result value [30, 31]. An eCDSS is intended to improve healthcare delivery by enhancing medical decisions with targeted clinical knowledge, patient information, and other health information [32].

This work describes the design, development, validation and preliminary evaluation of an eCDSS for supporting the management of dysglycaemia and diabetes in patients with severe mental illness. The eCDSS was designed to provide care professionals with screening reminders and clinically-relevant recommendations that are based on evidence-based clinical practice guidelines, and additional patient specific health information. Furthermore, this work evaluates the technical feasibility of implementing such an eCDSS in a secondary mental healthcare setting at a large secondary mental healthcare trust.

## Methods

The materials and methods that were used in this work are detailed in this section.

### Intervention development approach

**Fig. 1:**
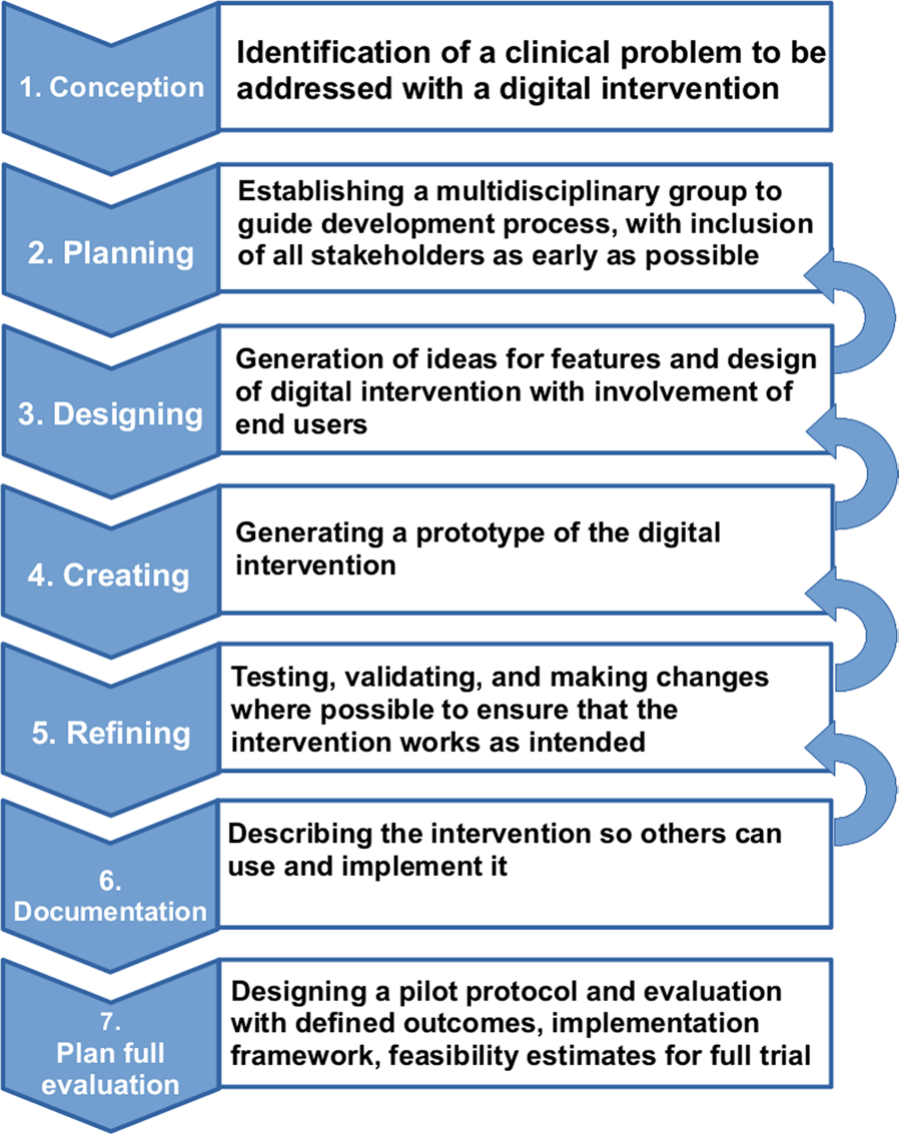
Intervention development approach

There are several approaches to developing interventions, programmes or innovations to improve health. The various approaches include a range of possible actions involved in intervention development, before undertaking any feasibility or piloting phase [33]. This work adopted a stepwise approach, with incremental and iterative steps covering a total of seven domains, as an overarching and guidance framework as shown in Fig. 1. An incremental and iterative approach was taken in order to allow continuous adaptation of the digital health intervention as the problem domain and deployment context become clearer. This technical feasibility evaluation work covered steps one to five, from *‘conception’* to *‘refining’*, of the overall intervention development approach.

### Scope and context

A Participatory Design approach was taken in this work to design and deploy a digital alerting system to support the clinical management of diabetes and dysglycaemia. Participatory Design entails the direct involvement of all stakeholders in the co-design of the technologies they use or that affect them [34]. The eCDSS was designed by a multidisciplinary team of patient and carer representatives, healthcare professionals, implementation scientists, health informaticians, and digital services professionals from South London and Maudsley National Health Service (NHS) Foundation Trust (SLaM), King’s College Hospital NHS Foundation Trust (KCH) and King’s College London. SLaM and KCH are large NHS Foundation Trusts that provide specialist services for mental health and acute care to South East London respectively. King’s College London is a University that carries out health informatics research. This work focused on designing, developing and evaluating the feasibility of an eCDSS for supporting the clinical management of dysglycaemia and diabetes amongst inpatients on acute psychiatry wards, a large proportion of whom are diagnosed with serious mental illness.

### Electronic health record retrieval and alerting with CogStack

A patient monitoring and alerting system was implemented in CogStack, a health information retrieval and curation platform. The CogStack platform leverages its wide-range of health information processing services, including modern data mining and alerting, that are orchestrated by a fault-tolerant batch processing framework that avails a configurable, searchable and patient-centric view of the EHR [35].

**Fig. 2:**
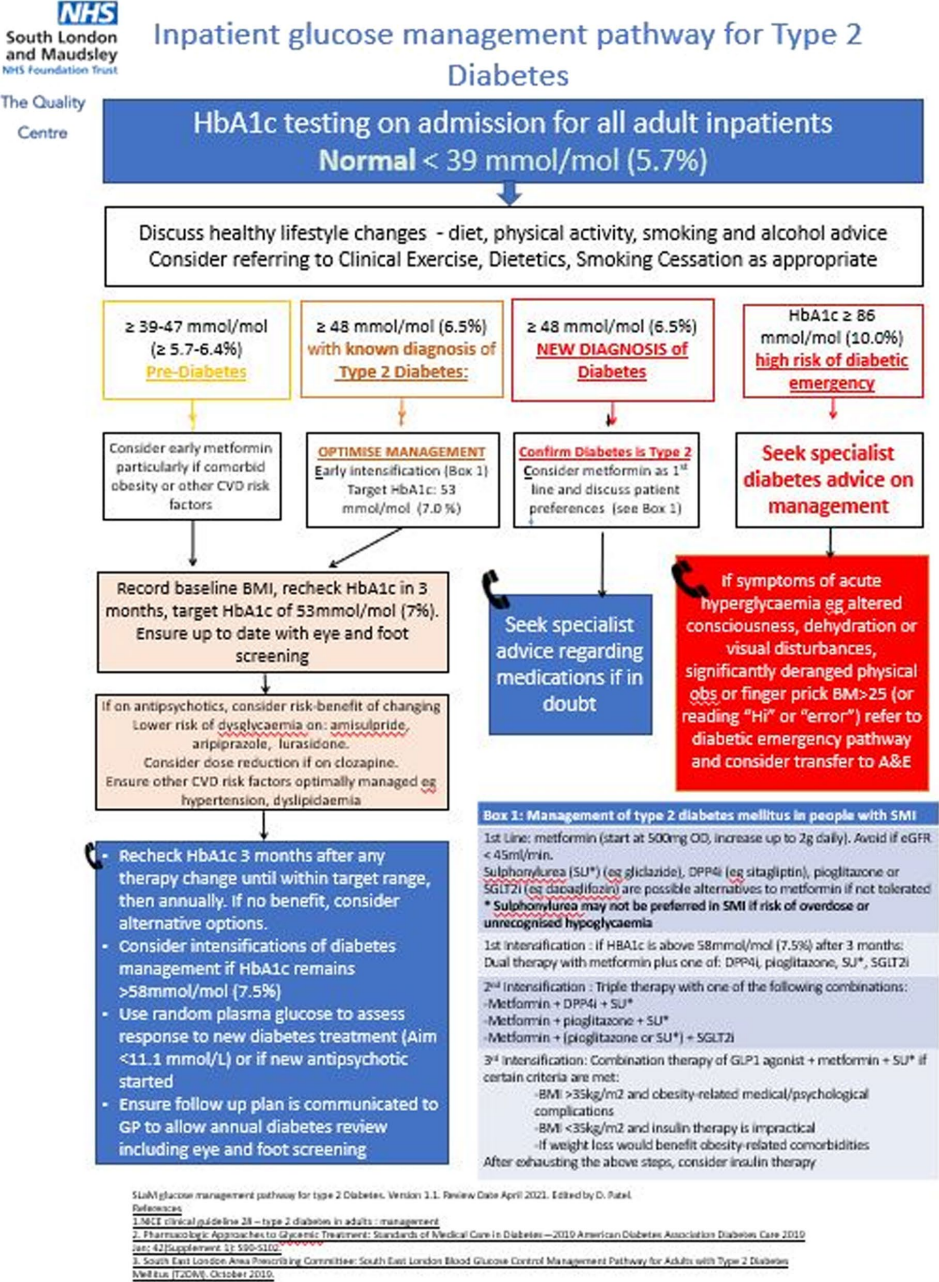
Paper clinical guidelines for type 2 diabetes

The patient monitoring and alerting solution was made up of three parts. The first part was a patient data pipeline that routinely pulls current and historical glycated haemoglobin (HbA1c) test result values for individual patients in near real time. The second part was a technical implementation of a clinical dysglycaemia and diabetes pathway. The SLaM-approved diabetes pathway is a combination of adapted National Institute for Health and Care Excellence (NICE) guidance [36] and clinical practice guidelines for physical health in Psychiatry [37] that was approved as standard of care at SLaM. These clinical guideline recommendations for Type 2 diabetes, as shown in Fig. 2, formed the basis of the digitalised clinical dysglycaemia and diabetes pathway. The technical implementation of the clinical pathway can be adjusted in future, to reflect changes in recommended second line medications for diabetes in SMI, namely glucagon-like peptide 1 (GLP-1) analogues [37]. The third part was a technical implementation and piloting of an automated alerting system that aims to ensure that clinicians are notified of at-risk patients and are prompted regarding potential evidence-based and patient-specific interventions initially via email before integration into the EHR system in a subsequent implementation.

#### Email alerting service for the diabetes management pathway

Email alerts contained SLaM approved guideline-based recommendations for clinician-led monitoring and management of dysglycaemia and known diabetes. These alerts were tailored to the individual patient based upon reported HbA1c test result values. The eCDSS triggers alerts based on the presence of new, historical or absent HbA1c pathology reports on the electronic health record (EHR) as per recommendations specified in the diabetes management guideline.

Key features of an eCDSS are likely to determine its future success in terms of adoption. To start with, alerts or reminders from an eCDSS should provide patient-specific recommendations, relevant for the clinical situation [28, 38]. An eCDSS should be intuitive and easy to use [28, 39]. User interactions and time to use the system should be kept to a minimum, and should be integrated with the EHR system where possible [28, 38]. Guideline recommendations should include explanations about the recommendation and be timed in line with clinicians’ individual daily working practices [28, 39]. Finally, an eCDSS should be designed in such a way that the user has choice and control over whether to use a guideline recommendations, giving the user continued judgement and autonomy of decision making [38].

With these factors in mind, information in the eCDSS alerts were planned to include one or more of the following:

1. Reminder regarding HbA1c monitoring where one has not been completed on admission, and/or when one is next due.
2. Latest HbA1c test result; previous HbA1c test results; and guidance on whether this currently meets threshold for diagnosis of pre-diabetes, diabetes, or decompensated (poorly controlled) diabetes, along with advice to determine whether a given patient has a known history of diabetes, as this will not be evident from an HbA1c level if well-controlled.
3. Prompting guideline-based lifestyle interventions—advice regarding exercise, diet and smoking cessation
4. Prompting guideline-based screening where indicated (such as: foot review, eye examination)
5. Signposting to guideline-based pharmacological interventions including details of appropriate first-line diabetes medication, how to approach intensification of medication to manage worsening glycaemic control in known cases of diabetes, discussion with specialist diabetes teams, information regarding antipsychotics with lower risk of dysglycaemia, and highlighting levels at which urgent referral to specialists in decompensated (severely poorly controlled) diabetes is indicated.
6. Links to relevant and more detailed clinical guidelines and medicines information on the NHS Trust staff intranet.

Automated electronic decision support was planned to be provided as an email sent to the NHS Trust email account addresses of the participating ward clinician(s).

### Evaluation setting and approach

**Fig. 3:**
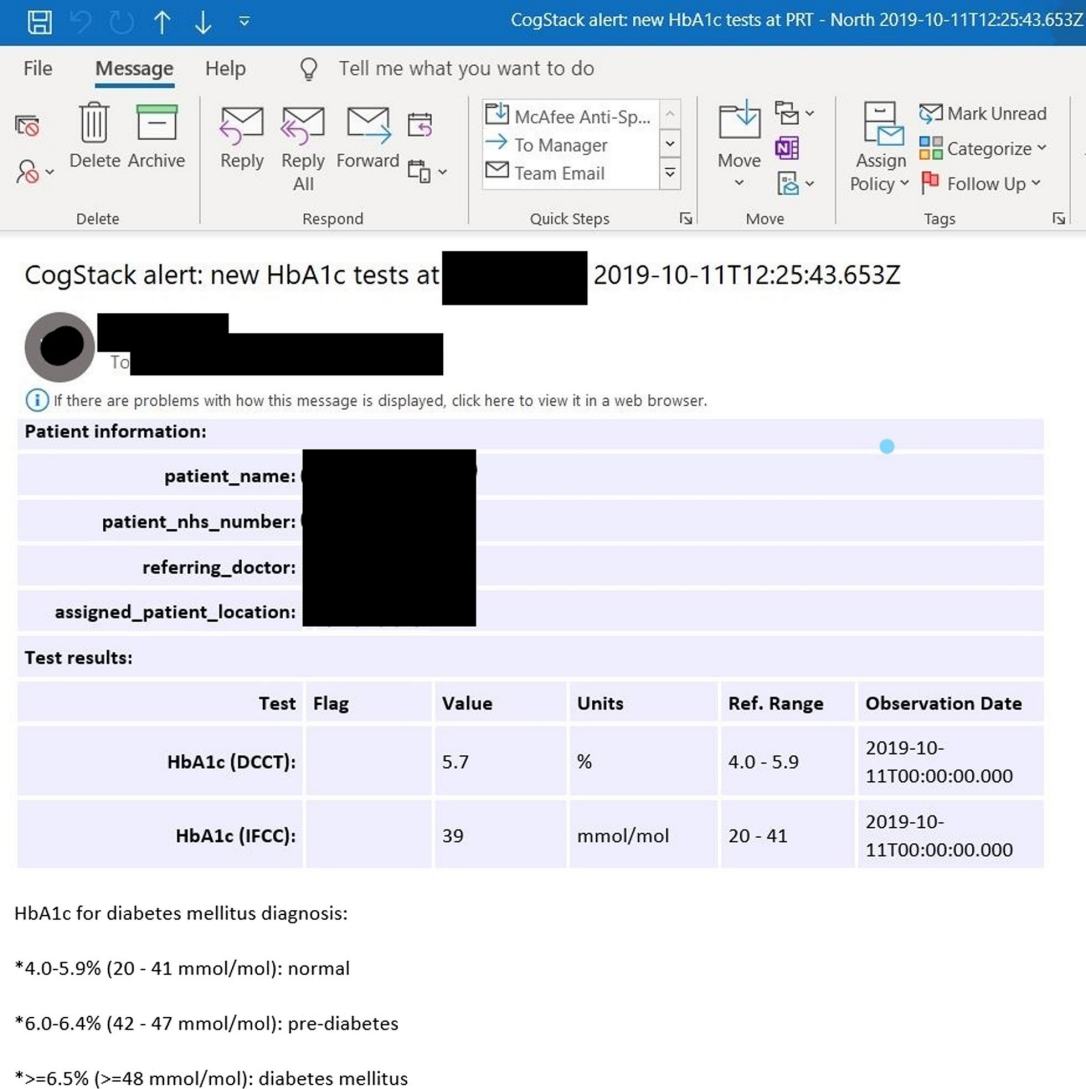
Screenshot of email alert (normal HbA1c)

**Fig. 4:**
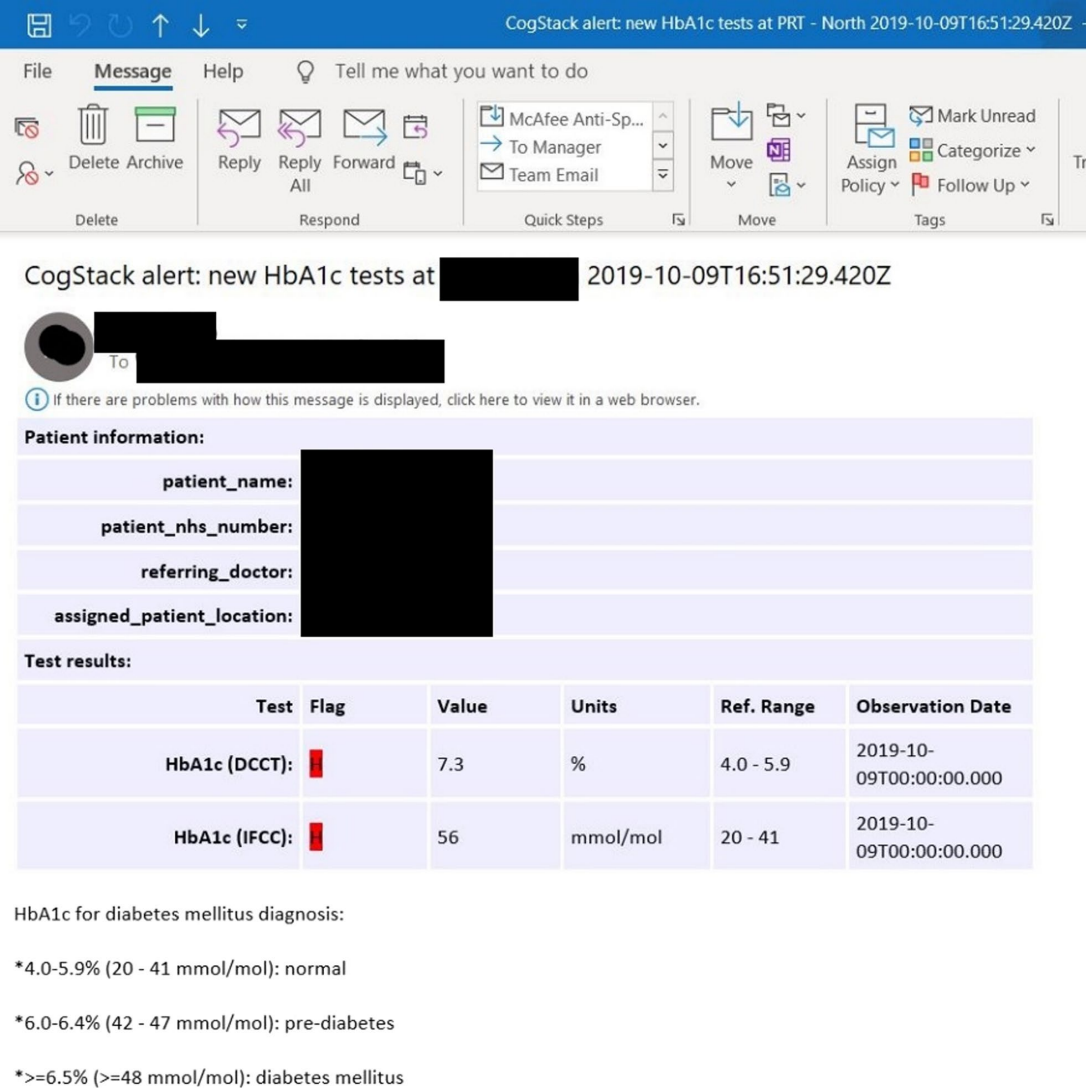
Screenshot of email alert (abnormal HbA1c flagged in red)

The initial technical evaluation was conducted with an outpatient community mental health team whereby the eCDSS was assessed for its ability to send email alerts containing patient name, patient NHS number (a unique patient’s identifier) and any related HbA1c test result data, along with reference values for HbA1c diagnostic thresholds, for the right patient and to the right clinician as shown in Fig. 3. Further testing was also conducted to assess the ability of the system to flag up an abnormal or raised HbA1c test result value in red as shown in Fig. 4.

Further detailed testing was then conducted in the same community mental health team outpatient sample, in order to assess the ability of the system to send email alerts to clinicians of patients in that sample. The individual alerts were manually cross-checked with EHR data against expected standards. The standards by which the monitoring and alerting system was initially assessed on were as follows:

1. System triggers alert for a patient once an HbA1c test result is available in the EHR
2. An alert is received by the correct clinician(s) in real time
3. An alert contains the correct clinical content and patient identifier such as correct HbA1c result, patient name and NHS number
4. An alert does not contain incorrect clinical information or incorrect identifiers such as incorrect patient name and NHS number
5. An alert contains information on HbA1c thresholds for diabetes and pre-diabetes diagnosis
6. An alert flags up an HbA1c test result in red if its value indicates that it is raised above threshold

In order to ensure that these standards were met, alerts were cross-referenced against their respective EHRs manually and the results were recorded.

Thereafter, the technical evaluation progressed to a more in-depth in silico validation of the monitoring and alerting intervention over a period of one month in three wards at SLaM. In silico-based engineering methods allow solutions to be investigated and evaluated in a computer-based simulated environment [40, 41]. The standards by which the eCDSS was evaluated *in silico* on were as follows:

1. System triggers one alert for patient on test ward once a new HbA1c result is reported on EHR
2. System triggers one alert for patient on test ward if no new HbA1c result is reported on EHR after 4 days post-admission
3. System triggers weekly alert reminders for patients on test ward if HbA1c result is not reported on EHR, after the initial reminder alert described in 2.
4. Alerts triggered include a summary of previous HbA1c reports if reported on EHR
5. Alert triggered for new HbA1c provides HbA1c value and details of which threshold range this fits into ie normal, pre-diabetes, diabetes, or severe dysglycaemia as per alerting algorithm
6. Alert provides prompts on recommended actions in line with clinical algorithm (Fig. 2)
7. Alert contains factually correct clinical and patient identifier information (correct HbA1c result, correct name, correct NHS number)
8. Alert does not contain incorrect clinical information or incorrect identifiers (name and NHS number)
9. Alerts are only triggered as per the algorithm (no additional or unexpected alerts triggered)
10. Alerts are not triggered once patients are discharged from ward.

### Legal, governance and ethical considerations

This work was approved by relevant NHS Trust governance and research bodies at SLaM. Ethical approval was granted from the NHS Health Research Authority (reference 285509). Furthermore, this work subscribes to the *‘code of conduct for data-driven health and care technology’* in order to account for ethical challenges associated with the use of data-driven technologies in the NHS and the wider health and care system [42].

## Results

A patient data pipeline that routinely examines the EHR in near real time was implemented in CogStack. The newly developed health information retrieval service routinely accesses HbA1c test result data and related patient information that enables monitoring and alerting of at-risk patients based on an approved diabetes management guideline at SLaM, a mental health NHS Foundation Trust. The prototype eCDSS was first evaluated over a period of one month. 22 alerts were generated as expected from a case load of 198 patients. Of the 22 alerts, nine alerts flagged HbA1c test results that were above threshold. The results of the evaluation against the set standards are summarised in Table 1.

**Table 1:** Results of the initial evaluation

		Expected (n)	Actual (n)
1.	Alert triggered	22	22
2.	Alert received by end-user	22	22
3.	Alert content provides clinically correct information on correct patient	22	22
4.	Alert content provides incorrect clinical information and/or incorrect patient identifier	0	0
5.	Alert contains HbA1c threshold information	22	22
6.	Alert flags up in red if HbA1c above threshold	9	9

**Table 2:** Results of the in silico validation

		Wards			Totals	
		*Ward X*	*Ward Y*	*Ward Z*	Expected (n)	Actual (n)
1.	System triggers one alert for patient on test ward once a new HbA1c result is reported in the EHR	11	7	14	32	32
2.	System triggers one alert for patient on test ward if no new HbA1c result is reported on EHR after 4 days post-admission	14	15	20	49	49
3.	System triggers weekly alert reminders for patients on test ward if HbA1c result is not reported on EHR, after the initial reminder alert described in (2)	7	4	9	20	20
4.	Alerts triggered include a summary of previous HbA1c reports if reported on EHR	32	26	43	101	101
5.	Alert triggered for new HbA1c provides HbA1c value and suggestion for which threshold range this fits into ie normal, pre-diabetes, diabetes, or severe dysglycaemia as per alerting algorithm	11	7	14	32	32
6.	Alert provides prompts on recommended actions in line with clinical algorithm	32	26	43	101	101
7.	Alert contains factually correct clinical and patient identifier information (correct HbA1c result, correct name, correct NHS number)	32	26	43	101	101
8.	Alert does not contain incorrect clinical information or incorrect identifiers (name and NHS number)	32	26	43	101	101
9.	Alerts are only triggered as per the algorithm (no additional or unexpected alerts triggered)	32	26	43	101	101
10.	Alerts are not triggered once patients are discharged from ward	0	0	0	0	0

The more in-depth and detailed in silico validation was then carried out over a period of one month in three wards. The results showed that all relevant alerts were generated, according to expected standard and inline with SLaM-approved clinical guideline recommendations. The alerts were generated as follows: a total of 32 alerts on the 18 bed *Ward X*, 26 alerts on the 19 bed *Ward Y*, and 43 alerts on the 23 bed *Ward Z* . The results from the in silico validation are summarised in Table 2.

## Discussion

The results from this technical evaluation of the physical health monitoring and alerting intervention, although limited in scope, revealed that it is technically feasible to deploy and evaluate a pragmatic guideline-based eCDSS in a secondary care mental health setting. To our knowledge, this is the first automated physical health monitoring and alerting intervention developed for use in a secondary care mental health setting. Evaluation and validation of eCDSSs for use in a clinical setting is time-consuming and its complexity can result in their slow adoption in practice [28]. To bridge this gap, the first step of the evaluation has covered the technical validation of the system. Further refinement, development and engineering of the eCDSS followed by clinical evaluation is thereafter needed to support and inform adoption into practice.

Integrating an eCDSS into a clinical workflow is not a trivial task. Digital health systems that are not accepted by their users cannot be expected to contribute to improving quality of care, hence facilitators and barriers to adoption need to be understood for successful implementation of novel tools. Gaining a good understanding of factors that affect adoption and integration of digital health tools in a clinician’s workflow could also serve as a basis for creating frameworks for delivering future impactful tools. Hence there have been calls for research to include evaluating eCDSS implementation for successful future scalability [39]. Understanding how best clinicians can be supported successfully at the point of care requires consideration of technological, clinical and socio-technical issues [28, 38].

Digital health evaluation frameworks that provide a technological, human and organizational fit are invaluable as they ensure that factors that may hinder adoption and use are identified as early as possible for rectification. The human, organization and technology-fit (HOT-fit) framework is one such framework that aims to assist researchers in conducting thorough evaluation studies of health information systems in a rigorous, systematic and continuous manner [43]. Recall the intervention development approach for this work that is described in the *Methodology:Intervention development* *approach* section and outlined in Fig. 1. The HOT-fit framework can be used to guide and inform a planned evaluation, under step seven of the intervention development approach, as this project progresses further.

The participatory approach taken in this work ensured that a wide-range of stakeholders including clinicians, informaticians, implementation scientists and patients were involved at all stages to ensure that the intervention is in a position to integrate as seamlessly as possible into the clinical workflow at SLaM. This approach further ensures that alert fatigue can be minimised. Previous studies have shown that clinicians overrode over 49% of alerts from an eCDSS due to low specificity, unnecessary workflow disruption, unclear information, and lack of user- and patient-context [28, 44]. It is therefore of paramount importance for eCDSS interventions to be aligned with the needs of all relevant stakeholders including its users in order to maximise its adoption and effectiveness in improving clinician adherence to guidelines for practice.

A major challenge in effective eCDSS implementation and maintenance is keeping its guideline-based clinical rules up to date which can be a time- and money-consuming task [38, 45]. The monitoring and alerting intervention from this work takes advantage of the microservices architecture of the CogStack platform which allows decision support services to be assembled from loosely-coupled microservices, thereby allowing easy maintenance of the clinical rules as the Trust-approved clinical pathways change over time. Therefore, if the clinical pathway as shown in Fig. 2 is updated in future, the alerting algorithm may be easily changed to mirror this.

## Conclusion

It is feasible to design and deploy a functional monitoring and alerting eCDSS in a secondary care setting for mental health for later clinical evaluation. Further work is required in order to meet the key requirements of the eCDSS namely that alerts are embedded within existing workflows as much as possible. In addition, before integrating into the clinical setting, further work is underway to ensure that the content of the alerts contains clinically-relevant information that is actionable, reliable, and readily understandable and meets NHS digital standards. Additional work is underway to generate and send the clinically-relevant alerts back into the EHR where they can be directly accessible from a dashboard in the EHR system by clinicians. Thereafter, a pilot acceptability and feasibility trial of the eCDSS will be conducted in a clinical setting. A further comprehensive evaluation of the implementation of the eCDSS will be conducted, whereby several implementation outcomes will be explored and evaluated alongside outcomes relating to feasibility, acceptability and impacts on care processes, based on established implementation science methods [46–48]. By describing and sharing the steps we will take from concept to clinical testing, we hope to provide useful insights to teams that are interested in building similar systems and starting out on their path to digital health innovation, bridging the gap between technology development and implementation in health care settings. An eCDSS with a monitoring and alerting mechanism has the potential to improve clinician-led management of diabetes and dysglycaemia in mental health settings. If found to be feasible and acceptable in a pilot trial, then in combination with results of the implementation evaluation, the system can be refined and potential problems with future successful implementation addressed. A larger and more definitive effectiveness trial can then be conducted to assess the impact of the eCDSS on clinical outcomes in diabetes, and to inform its application and scalability for other conditions such as atrial fibrillation, hypertension and hypercholesterolaemia in wider mental healthcare settings.

## Data Availability

The datasets generated and/or analysed during the current study are not publicly available due SLaM’s policy restrictions on sharing electronic patient records but are available from the corresponding author on reasonable request.
